# Inexpensive Systemic Inflammatory Biomarkers in Ovarian Cancer: An Umbrella Systematic Review of 17 Prognostic Meta-Analyses

**DOI:** 10.3389/fonc.2021.694821

**Published:** 2021-09-23

**Authors:** Khalid El Bairi, Ouissam Al Jarroudi, Said Afqir

**Affiliations:** ^1^ Department of Medical Oncology, Mohammed VI University Hospital, Oujda, Morocco; ^2^ Faculty of Medicine and Pharmacy, Mohammed Ist University, Oujda, Morocco

**Keywords:** ovarian cancer, lymphocyte-to-monocyte, neutrophil-to-lymphocyte, platelet-to-lymphocyte, systemic inflammatory biomarkers, umbrella review

## Abstract

The association of several inflammation-based biomarkers [lymphocyte-to-monocyte, neutrophil-to-lymphocyte, and platelet-to-lymphocyte ratios (LMR, NLR, and PLR, respectively)] with the survival of epithelial ovarian cancer (EOC) patients has been extensively investigated in several systematic reviews and meta-analyses (MAs) of observational studies. The aim of this umbrella systematic review is to appraise all available results in published MAs that explored the association between these biomarkers and EOC outcomes. An umbrella systematic review of the current evidence for systemic inflammatory biomarkers in the peripheral blood of EOC patients was performed by searching several databases including PubMed/Medline and Web of Science. The quality of the MAs was appraised using the AMSTAR-2 tool as well as other qualitative criteria. The evidence was graded from convincing (Class I) to weak (Class IV). Our umbrella review appraised 17 MAs of retrospective studies (range: 7–16) with a number of enrolled patients ranging from 1,636 to 4,910 patients in each MA. All these MAs demonstrated that pretreatment high NLR and PLR, as well as low LMR, were independent predictors of poor overall survival and progression-free survival in EOC. Nearly all published MAs were conducted by Chinese researchers (16/17) and were redundant in their character. Another issue in these MAs is the absence of prior PROSPERO database registration as well as the earlier exclusion of the gray literature. On the other hand, Preferred Reporting Items for Systematic Reviews and Meta-Analyses (PRISMA) and Meta-analyses Of Observational Studies in Epidemiology (MOOSE)-based reporting guidelines were used in nine out of the 17 MAs. A good number of MAs have transparently provided funding acknowledgment. The AMSTAR-2-based assessment showed low quality in 11 out of the 17 reviewed MAs. This negative rating was largely due to the absence of critical domains. Finally, all evaluated MAs were rated as Class III or IV (suggestive and weak, respectively). Despite the power of MAs in increasing sampling and precision, the quality of the current non-randomized evidence on this topic is still weak.

**Systematic Review Registration:**

PROSPERO, identifier CRD42020201493.

## Introduction

There is a remarkable trend in modern oncology to implement accurate biomarkers for predicting therapy response, prognosis, and survival of cancer patients. The advent of biomarker-based targeted agents such as poly-(ADP-ribose)-polymerase (PARP) inhibitors and immune-checkpoint blockers and several molecular signatures for patients’ prognostic stratification was successfully introduced into the management of various gynecologic cancers. A number of these drug targets and their biomarkers were discovered based on the “Hallmarks of Cancer” principles, which have deeply changed our understanding of this disease and advanced oncology toward precision medicine (1–3). Inflammation is one of these hallmarks described in epithelial ovarian cancer (EOC) (4). Remarkably, systemic inflammation is well reported to be involved in carcinogenesis by driving tumor initiation, growth, progression, and metastasis (5). A variety of inflammation-derived biomarkers were explored in solid cancers and showed predictive power for prognosis (6, 7). In EOC, an important number of circulating blood-based and inexpensive inflammatory biomarkers were recently suggested to predict outcomes. This is essentially based on pretreatment complete blood count including lymphocyte-to-monocyte, neutrophil-to-lymphocyte, and platelet-to-lymphocyte ratios (LMR, NLR, and PLR, respectively) (8–10). Their independent predictive value of survival in EOC was assessed in multiple systematic reviews and meta-analyses (MAs) to increase sample size and power. The findings of these pooled analyses demonstrated that low LMR predicts reduced overall survival (OS) and progression-free survival (PFS) (8, 11). Moreover, low LMR is associated with advanced International Federation of Gynecology and Obstetrics (FIGO) stages, malignant ascites, lymph node metastasis, chemotherapy resistance, and high levels of cancer antigen 125 (CA-125) (8, 11). Similarly, high NLR was also revealed to be associated with advanced grade and stage, bilateral tumors, and EOC risk factors as well as worse survival outcomes (10, 12). On the other hand, high PLR negatively impacts both the OS and PFS in the same setting (13, 14). This umbrella review of systematic reviews and MAs, which is a recently developed article type, was conducted to revisit and critically appraise the quality of these published MAs and provide an updated examination of the current evidence on this topic using the assessment of multiple systematic reviews (AMSTAR-2) tool (15).

## Methods

### Umbrella Systematic Review and Search Strategy

**Figure 1:**
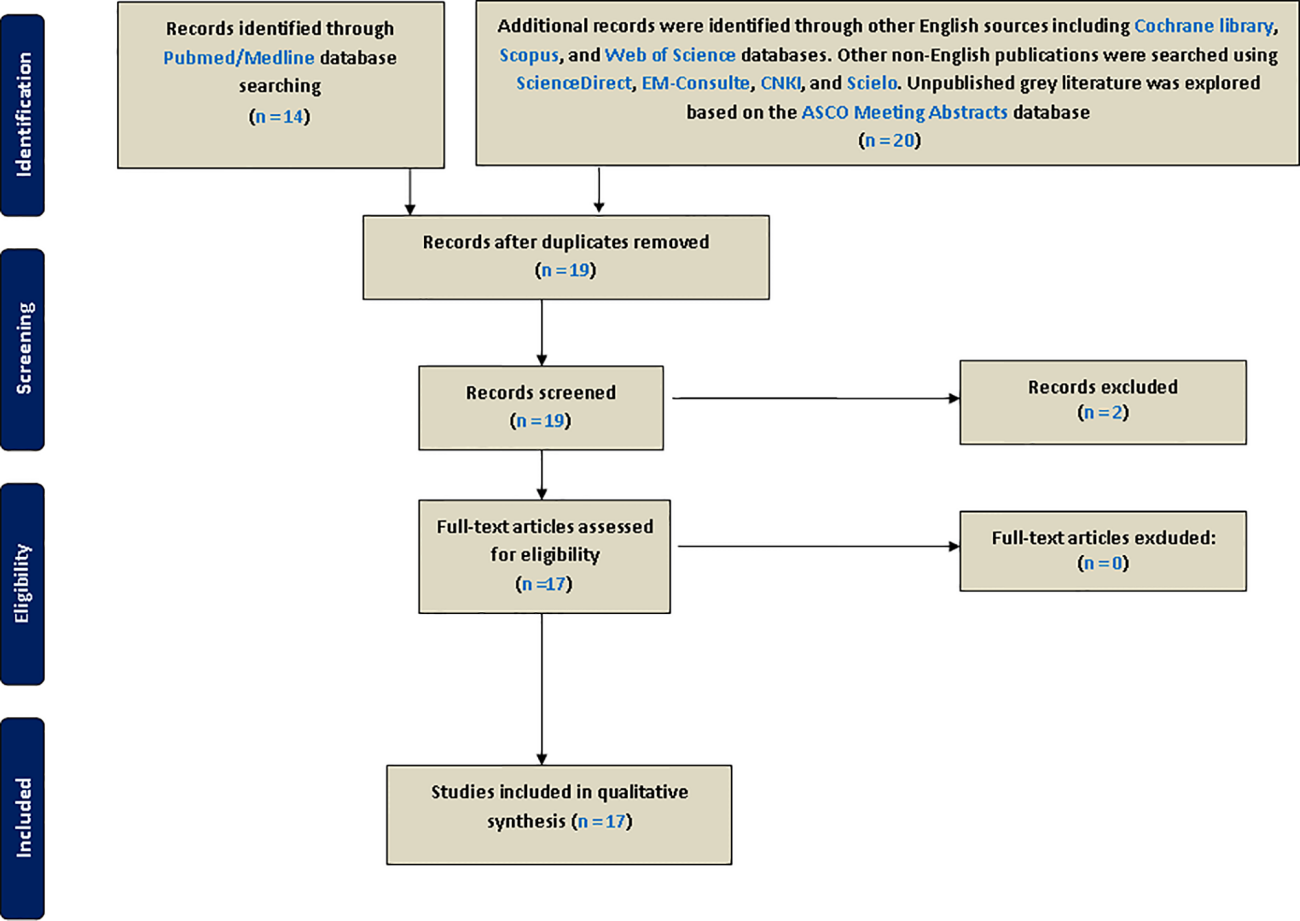
Flow diagram of article selection.

As recommended by international guidelines for best practice when conducting systematic reviews, this umbrella study was registered in PROSPERO’s International Prospective Register of Systematic Reviews (reg. number: CRD42020201493). This initiative is an international database of the York University (UK) aiming to prospectively register systematic reviews and MAs in various aspects of health-related outcomes to limit redundancy, reduce reporting bias, and promote transparency (16). We conducted this umbrella review by systematically searching for previously published systematic reviews and MAs in which inflammation-based biomarkers including LMR, NLR, and PLR have been described in EOC. This article type can be easily searchable on available bibliographic databases using automatic filters. First, PubMed/Medline, which covers most medical journals, were searched for relevant systematic reviews and MAs published in English from the beginning of article indexing to August 1, 2020, by using the following keywords: [(systematic review) OR meta-analysis] AND (ovarian cancer) AND (((lymphocyte-to-monocyte ratio) OR neutrophil-to-lymphocyte ratio) OR platelet-to-lymphocyte ratio). Besides, we also used cross-referencing to find other MAs in the references of selected eligible articles. Supplementary searches of the English literature were performed on the Cochrane Library, Scopus, and Web of Science databases. To cover other non-English publications and limit language bias, we searched ScienceDirect (https://www.sciencedirect.com/) and EM-Consulte (https://www.em-consulte.com/produits/traites) for French, CNKI (China National Knowledge Infrastructure) for Chinese (http://oversea.cnki.net/), and SciELO for Spanish (https://www.scielo.br/). Moreover, unpublished gray literature was explored based on the ASCO Meeting Abstracts database (https://ascopubs.org/jco/meeting). Article titles and abstracts were independently screened and reviewed before inclusion by two reviewers (KE and OA). Eligible MAs were screened for full-text and reviewed (**Figure 1**). In case of any disagreement, a consensus was reached after discussion between the authors.

### Eligibility Criteria and Data Extraction

Articles were selected in our umbrella review only if they met the following mandatory inclusion criteria: a) systematic reviews or systematic reviews with MAs of observational or interventional studies; b) the study population enrolling EOC patients only; c) in case of reviews including other solid cancers, EOC must be studied separately in a subgroup analysis based on tumor type; d) selected reviews with pooled findings on at least one of the three biomarkers studied; and finally e) articles were included if they contained prognostic extractable data on survival outcomes (OS and PFS). Other article types including original clinical studies and narrative reviews were excluded. However, they were rarely consulted to find other MAs cited in their reference lists. Conference abstracts communicated at American Society of Clinical Oncology (ASCO) meetings were also searched to find unpublished MAs. Data extraction was then conducted by two reviewers (first by KE and verified by OA). Relevant general characteristics of selected studies encompassing the following were extracted and summarized: author/year, biomarkers studied, journal, country, number of included studies and their design, patients enrollment, endpoints, pooled hazard ratio (HR) and their corresponding confidence intervals with *p*-values, heterogeneity (I^2^ metric) and related *p*-value, evaluation of the source of heterogeneity, *p*-values of publication bias tests, PROSPERO registration, use of reporting guidelines, use of Newcastle–Ottawa Scale (NOS), subgroup analysis, the search of gray literature, and finally funding. Forest plotting of overall effects based on HRs and their confidence intervals was performed using the extracted data from each MA.

### Assessment of Methodological Quality in Included Meta-Analyses

The quality of the MA methodology was investigated using the AMSTAR-2 checklist (https://amstar.ca/Amstar_Checklist.php) (15). This tool is reliable for assessing the quality of systematic reviews and MAs of human observational and interventional studies. This revised instrument contains 16 items in total including seven critical questions (Q2, Q4, Q7, Q9, Q11, Q13, and Q15), and it is not intended to generate an overall score. Based on these items, the quality of the systematic reviews and MAs is categorized into high, moderate, low, or critically low (15). The AMSTAR-2-based assessment was first performed by KE and independently reviewed by OA with disagreements sorted by consensus.

### Grading the Evidence

The evidence was categorized into Class I (convincing), Class II (highly suggestive), Class III (suggestive), or Class IV (weak evidence), following the previously described grading scheme (17). The criteria were modified to include the design of included studies instead of the excess of significance test because of the low power of this statistic. Moreover, the excess of significance evaluation is not currently recommended by the Cochrane guide of systematic reviews of interventions (18). The criteria for each class are described as follows: A) Class I (strong evidence): prospective design of included studies, *p-*value of overall effect <10^−6^, I^2^ < 50%, calculated 95% prediction interval excluding the null value, sample size >1,000 cases, and no evidence of small-study effects (publication bias using Egger test). B) Class II (highly suggestive): prospective design of included studies, *p-*value of overall effect <10^−6^, and sample size >1,000 cases. C) Class III (suggestive): retrospective design of included studies, *p-*value of overall effect <10^−3^, and sample size >1,000 cases. D) Class IV (weak evidence): retrospective design of included studies and *p-*value <0.05. MAs that did not report *p*-values of overall effects were not rated. Also, we decided to downgrade all MAs to Classes III and IV when they included evidence from retrospective studies.

## Results

### General Characteristics of Included Systematic Reviews and Meta-Analyses

Overall, a total number of 17 MAs were found and analyzed, encompassing nine items for NLR, six for PLR, and four for LMR (**Table 1**). Moreover, 15/17 eligible MAs have investigated the prognostic value of single biomarkers, and only two have incorporated two biomarkers (14, 24). The included articles were published between 2017 and 2020, and they were all MAs of retrospective studies.

**Table 1:** General characteristics of included systematic reviews and meta-analyses.

Author/year	Biomarkers studied	Journal	Country	N	Type of included studies	Patient enrollment
Cai et al., 2020 (19)	LMR	*Medicine*	China	9	Retrospective	2,809
Yin et al., 2019 (20)	NLR	*Medicine*	China	10	Retrospective	2,919
Gong et al., 2019 (8)	LMR	*J Ovarian Res*	China	8	Retrospective	2,259
Gao et al., 2019 (21)	LMR	*Cancer Manag Res*	China	12	Retrospective	3,346
Jiang et al., 2019 (22)	PLR	*Arch Gynecol Obstet*	China	10	Retrospective	2,490
Lu et al., 2019 (11)	LMR	*Medicine*	China	7	Retrospective	2,343
Tian et al., 2018 (13)	PLR	*Eur J Clin Invest*	China	11	Retrospective	3,574
Xu et al., 2018 (23)	PLR	*Transl Cancer Res*	China	8	Retrospective	1,636
Zhao et al., 2018 (24)	NLR and PLR	*Arch Gynecol Obstet*	China	13	Retrospective	3,467
Chen et al., 2018 (25)	NLR	*Technol Cancer Res Treat*	China	12	Retrospective	4,064
Zhu et al., 2018 (14)	NLR and PLR	*BMC Cancer*	China	10	Retrospective	2,919
Chen et al., 2017 (26)	NLR	*Biomed Res Int*	China	11	Retrospective	2,892
Ma et al., 2017 (27)	PLR	*Climacteric*	China	12	Retrospective	2,340
Huang et al., 2017 (28)	NLR	*Cell Physiol Biochem*	China	12	Retrospective	3,854
Yang et al., 2017 (29)	NLR	*Oncotarget*	China	12	Retrospective	3,154
Ethier et al., 2017 (10)	NLR	*Gynecol Oncol*	Canada	12	Retrospective	3,376
Zhou et al., 2017 (30)	NLR	*Oncotarget*	China	16	Retrospective	4,910

LMR, lymphocyte-to-monocyte ratio; NLR, neutrophil-to-lymphocyte ratio; N, number of included studies; PLR, platelet-to-lymphocyte ratio.

In 2017, five MAs on the NLR biomarker were published. Similarly, three other redundant MAs, on the same biomarker, were published in 2018. Four other MAs on PLR and three on LMR were published in 2018 and 2019, respectively. The number of included articles in each MA ranged from 7 to 16 with a number of enrolled EOC patients between 1,636 (23) and 4,910 (30). Notably, most of the published MAs were from China (16/17), with only one found article from Canadian researchers (10).

#### Meta-Analyses on the Lymphocyte-to-Monocyte Ratio

**Figure 2:**
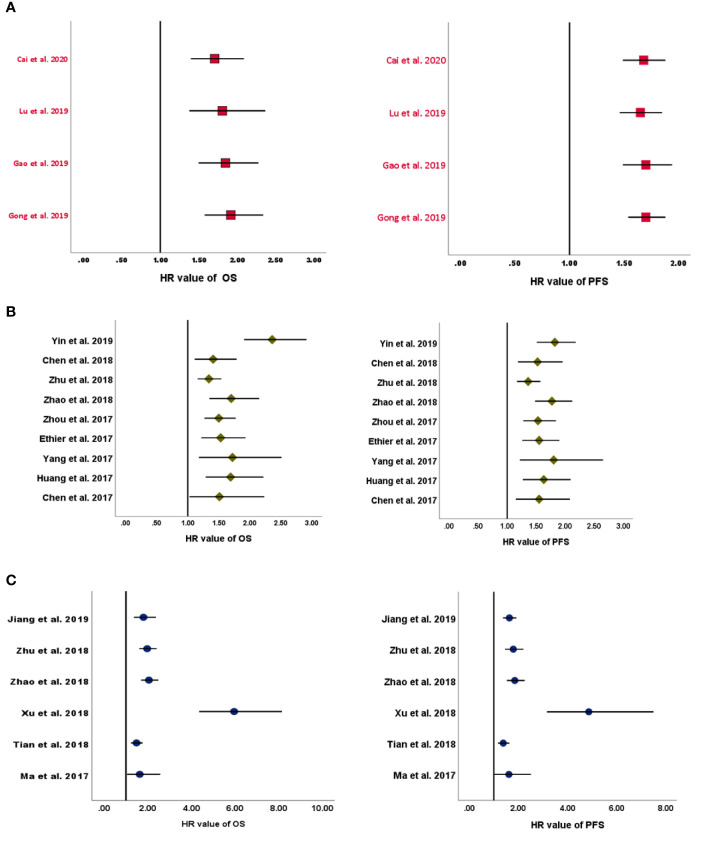
Forest plots of pooled HRs for **(A)** LMR, **(B)** NLR, and **(C)** PLR. HRs, hazard ratios; LMR, lymphocyte-to-monocyte ratio; NLR, neutrophil-to-lymphocyte ratio; PLR, platelet-to-lymphocyte ratio.

In the four MAs on LMR (**Table 2**), OS and PFS were the endpoints used. The results of the pooled studies in all the MAs demonstrated that low LMR significantly predicted poor survival outcomes in EOC [for both OS (HR: 1.71–1.92) and PFS (HR: 1.70–1.65)] (**Figure 2A**). Heterogeneity was noticeable for OS (>65%, *p* < 5%) but minor for PFS in three studies and moderate in one MA (>45%; *p* = 0.09) (8). All these MAs conducted a sensitivity analysis to evaluate the source of heterogeneity. Yet only one MA used meta-regression (21). Accordingly, the final pooled HRs were stable in three studies (8, 11, 21). In the study of Lu et al., Tang et al. (31), Wang et al. (32), and Li et al. (33) were found to contribute to the observed heterogeneity. In another MA, exclusion of studies did not reduce heterogeneity and was above 50% (19). The publication bias was assessed based on Egger’s and Begg’s regression and rank correlation tests in three MAs. One MA stated the search of publication bias, but this was not evaluated (8). In addition, one MA did not conduct statistical analysis for PFS, as the conditions were not met (11). No significant findings were revealed by these two statistical methods, suggesting no publication bias for OS and PFS.

**Table 2:** Outcomes, heterogeneity, and publication bias in included meta-analyses.

Author/year	Biomarkers	Endpoint	Pooled HR[95% CI]; *p*-value	Heterogeneity (I^2^); *p*-value	Source of heterogeneity evaluated?	Results of the sensitivity analysis	*p*-Value of publication bias tests
**Lymphocyte-to-monocyte ratio**							
Cai et al., 2020 (19)	LMR	OS and PFS	**-OS:** 1.71 [1.40–2.09]; *p* < 0.001 **-PFS:** 1.68 [1.49–1.88]; *p* < 0.001	**-OS:** 69.8%; *p* = 0.001 **-PFS:** 0.2%; *p* = 0.405	Yes (sensitivity analysis)	-The heterogeneity was still above 50% when excluding each study -The exclusion of the study of Tang et al. (31) decreased the heterogeneity to the lowest value (51.9%)	**-OS:** *p* = 0.348 **-PFS:** *p* = 0.806 (Begg’s test)
Gong et al., 2019 (8)	LMR	OS and PFS	-**OS:** 1.92 [1.58–2.34]; *p* < 0.001 -**PFS:** 1.70 [1.54–1.88]; *p* < 0.001	-**OS:** 70%; *p* = 0.001 -**PFS:** 48%; *p* = 0.09	Yes (sensitivity analysis)	-None of the included studies substantially altered final results	Stated but not assessed
Gao et al., 2019 (21)	LMR	OS and PFS	-**OS:** 1.85 [1.50–2.28]; *p* < 0.001 -**PFS:** 1.70 [1.49–1.94]; *p* < 0.001	-**OS:** 76.5%; *p* < 0.001 -**PFS:** 24.4%; *p* = 0.234	Yes (sensitivity analysis and meta-regression)	-None of the included studies substantially altered final results	-**OS:** Egger’s test: *p* = 0.732 Begg’s test: *p* = 0.272 -**PFS:** Egger’s test: p=1.000 Begg’s test: *p* = 0.887
Lu et al., 2019 (11)	LMR	OS and PFS	-**OS:** 1.81 [1.38–2.37]; *p* < 0.01 -**PFS:** 1.65 [1.46–1.85]; *p* < 0.01	-**OS:** 78%; *p* = 0.0001 -**PFS:** 5%; *p* = 0.35	Yes (sensitivity analysis)	-The pooled HRs were not affected when excluding studies -Tang et al. (31), Wang et al. (32), and Li et al. (33) were the main sources of heterogeneity	**-OS:** Begg’s test: *p* = 0.368 Egger’s test: *p* = 0.185 **-PFS:** conditions not met to conduct statistical analysis
**Neutrophil-to-lymphocyte ratio**							
Yin et al., 2019 (20)	NLR	OS and PFS	-**OS:** 2.36 [1.91–2.91]; *p* < 0 .001 -**PFS:** 1.82 [1.51–2.18]; *p* < 0.001	-**OS:** 70%; *p* = 0.0004 -**PFS:** 36%; *p* = 0.12	Yes (sensitivity analysis)	-None of the included studies had an excessive influence on the stability of the final HR	-**OS and PFS:** funnel plotting only, no *p*-values provided for Egger’s and Begg’s tests
Zhao et al., 2018 (24)	NLR	OS and PFS	-**OS:** 1.70 [1.35–2.15] -**PFS:** 1.77 [1.48–2.12] Note: *p*-values of the overall effect were not provided	-**OS:** 64.4%; *p* = 0.001 -**PFS:** 43.2%; *p* = 0.062	Yes (sensitivity analysis)	-None of the included studies substantially altered the final results of OS -Exclusion of the study of Feng et al. (2016) (34) decreased the heterogeneity to 0% with stable HR for PFS	-**OS:** Begg’s test: *p* = 0.150 Egger’s test: *p* = 0.052 -**PFS:** Begg’s test: *p* = 0.755 Egger’s test: *p* = 0.015
Zhu et al., 2018 (14)	NLR	OS and PFS	-**OS:** 1.34 [1.16–1.54] -**PFS:** 1.36 [1.17–1.57] Note: *p*-values of the overall effect were not provided	-**OS:** 88.5%; *p* = 0.000 -**PFS:** 93.8%; *p* = 0.000	Yes (sensitivity analysis)	-None of the included studies had an excessive influence on the stability of the final HR	Only funnel plots were provided
Chen et al., 2018^κ^ (25)	NLR	OS and PFS	-**OS:** 1.64 [1.41–1.90]; *p* = 0.000 -**PFS:** 1.61 [1.42–1.83]; *p* = 0.000	-**OS:** 88.9%; *p* = 0.000 -**PFS:** 81.8%; *p* = 0.000	Yes (sensitivity analysis)	-The final combined results were not affected considerably -Related data were not shown by the authors	-**OS:** Egger’s test: *p* = 0.161 -**PFS:** Egger’s test: *p* = 0.230
Chen et al., 2017 (26)	NLR	OS and PFS	-**OS:** 1.51 [1.03–2.23]; *p* = 0.04 -**PFS:** 1.55 [1.15–2.08]; *p* = 0.004	-**OS:** 85%; *p* < 0.00001 -**PFS:** 61%; *p* = 0.03	Yes (sensitivity analysis)	-The final combined results were not affected considerably	Begg’s test: *p* = 0.175 Egger’s test: *p* = 0.160
Huang et al., 2017 (28)	NLR	OS and PFS	-**OS:** 1.69 [1.29–2.22] -**PFS:** 1.63 [1.27–2.09] Note: *p*-values of the overall effect were not provided	-**OS:** 68.3%; *p* < 0.001 -**PFS:** 56.6%; *p* = 0.024	Yes (sensitivity analysis)	-The final combined results were not affected considerably	-**OS:** Egger’s test: *p* = 0.061 Beggar’s test**^†^**: *p* = 0.150 -**PFS:** Egger’s test: *p* = 0.203 Beggar’s test**^†^**: *p* = 0.536
Yang et al., 2017 (29)	NLR	OS and PFS	-**OS:** 1.72 [1.18–2.51] -**PFS:** 1.80 [1.22–2.65] Note: *p*-values of the overall effect were not provided	-**OS:** 73.5%; *p* = 0.000 -**PFS:** 79.1%; *p* = 0.000	Yes (sensitivity analysis)	-The final combined results were not affected considerably	-**OS:** Egger’s test: *p* = 0.16 Begg’s test: *p* = 0.15 -**PFS:** Egger’s test: *p* = 0.26 Begg’s test: *p* = 0.55
Ethier et al., 2017 (10)	NLR	OS and EFS	-**OS:** 1.53 [1.22–1.93]; *p* < 0.001 -**EFS:** 1.55 [1.26–1.90]; *p* < 0.001	-**OS:** 74%; *p* < 0.001 -**EFS:** 66%; *p* = 0.003	Yes (sensitivity analysis and meta-regression)	-Exclusion of studies did not affect the heterogeneity results	Only funnel plots were provided
Zhou et al., 2017 (30)	NLR	OS and PFS	-**OS:** 1.50 [1.27–1.77]; *p* < 0.001 -**PFS:** 1.53 [1.28–1.84]; *p* < 0.001	-**OS:** 80.2%; *p* < 0.001 -**PFS:** 85.2%; *p* < 0.001	Yes (sensitivity analysis and meta-regression)	-The final combined results were not affected considerably	-**OS:** Egger’s test: *p* < 0.000 -**PFS:** Egger’s test: *p* = 0.001
**Platelet-to-lymphocyte ratio**							
Jiang et al., 2019 (22)	PLR	OS and PFS	-**OS:** 1.80 [1.37–2.37]; *p* = 0.000 -**PFS:** 1.63 [1.38–1.91]; *p* = 0.000	-**OS:** 70.7%; *p* = 0.001 -**PFS:** 15.9%; *p* = 0.312	Yes (sensitivity analysis)	-Exclusion of the study of Li et al. (2017) (33) decreased the heterogeneity significantly for OS -The final combined results were not affected considerably for PFS	Assessed for both OS and PFS by Begg’s and Egger’s tests but included other cancer types
Tian et al., 2018 (13)	PLR	OS and PFS	-**OS:** 1.48 [1.24–1.76]; *p* < 0.001 -**PFS:** 1.38 [1.17–1.63]; *p* < 0.001	-**OS:** 89%; *p* < 0.001 -**PFS:** 89%; *p* < 0.001	Yes (sensitivity analysis)	-The pooled HRs were not affected when excluding studies	Funnel plotting only
Xu et al., 2018 (23)	PLR	OS and PFS	-**OS:** 5.95 [4.35–8.14]; *p* = 0.000 -**PFS:** 4.86 [3.16–7.49]; *p* < 0.001	-**OS:** 0%; *p* = 0.872 -**PFS:** 43.4%; *p* = 0.132	Yes (sensitivity analysis)	-The final results were the same after the sensitivity analysis	-**OS:** Egger’s and Begg’s tests: *p* = 0.269 -**PFS:** Egger’s and Begg’s tests: *p* = 0.243
Zhao et al., 2018 (24)	PLR	OS and PFS	-**OS:** 2.05 [1.70–2.48] -**PFS:** 1.85 [1.53–2.25] Note: *p*-values of the overall effect were not provided	-**OS:** 0%; *p* = 0.663 -**PFS:** 0%; *p* = 0.942	Yes (sensitivity analysis)	-No heterogeneity was detected for both OS and PFS	-**OS:** Begg’s test: *p* = 0.452 Egger’s test: *p* = 0.558 -**PFS:** Begg’s test: *p* = 0.221 Egger’s test: *p* = 0.255
Zhu et al., 2018 (14)	PLR	OS and PFS	-**OS:** 1.97 [1.61–2.40] -**PFS:** 1.79 [1.46–2.20] Note: *p*-values of the overall effect were not provided	-**OS:** 75%; *p* = 0.001 -**PFS:** 81.2%; *p* = 0.000	Yes (sensitivity analysis)	-None of the included studies had an excessive influence on the stability of the final HR	Funnel plotting only
Ma et al., 2017 (27)	PLR	OS and PFS	-**OS:** 1.63 [1.05–2.56]; *p* < 0.01 -**PFS:** 1.61 [1.03–2.51]; *p* < 0.01	-**OS:** 93%; *p* < 0.00001 -**PFS:** 89%; *p* < 0.00001	No	-Sensitivity analysis was not conducted	Funnel plotting only

EFS, event-free survival; OS, overall survival; PFS, progression-free survival; HR, hazard ratio.

^†^We checked this publication bias statistical test, and we did not find it in the literature. This was also confirmed by a statistician. We hope this was a typo.

^κ^The data of this publication were recently updated; see here: doi: http://www.10.1177/1533033820973812.

#### Meta-Analyses on the Neutrophil-to-Lymphocyte Ratio

All included MAs (**Table 2**) used OS and PFS as primary endpoints except for one study that used event-free survival (EFS) to encompass disease-free survival in addition to PFS (10). The HR of the overall effect ranged from 1.34 to 2.36 for OS and 1.523 to 1.82 for PFS, which was significant in all studies. Therefore, the pooled HR showed that high NLR at baseline was significantly associated with worse OS and PFS in EOC (**Figure 2B**). *p*-Values of the pooled overall effect were not provided in two MAs (14, 24). Substantial and significant heterogeneity was found in all MAs of OS (>60%). Similarly, MAs of PFS and EFS had also significant heterogeneity, except for one study in which heterogeneity was moderate (36%) (20). A sensitivity analysis was performed in all MAs. Besides, two studies conducted meta-regression in addition to the sensitivity analysis (10, 30). It seems that heterogeneity did not affect the final combined results considerably in most MAs. Nevertheless, when the study of Feng et al. (34) was excluded from the MA of Zhao et al. (24), the heterogeneity was reduced to 0%, with stable HR for PFS. Strangely, related data of a sensitivity analysis in one MA were not shown by the authors (25). For publication bias, both Egger’s and Begg’s statistical tests were used. Funnel plotting only was used in three studies (10, 14, 20). Publication bias was absent in five MAs (14, 20, 25, 26, 29) and detected in three others (10, 24, 30). In another MA (28), the *p*-values of Egger’s and Begg’s tests were discordant; and the authors stated the low probability of publication bias.

#### Meta-Analyses on the Platelet-to-Lymphocyte Ratio

OS and PFS were the endpoints used in all included MAs (**Table 2**). The HR of the overall effect ranged from 1.48 to 5.95 for OS and 1.38 to 4.86 for PFS, which was significant in all studies. Therefore, high pretreatment PLR was a negative prognostic biomarker for OS and PFS in EOC (**Figure 2C**). Of note, the *p*-values of the overall effect were not provided in two MAs (14, 24). Heterogeneity was substantial in four MAs of OS (>70%, *p* < 5%) (13, 14, 22, 27) and in three MAs of PFS (>80%, *p* < 5%) (13, 14, 27); moderate in one MA of PFS (43.4%) (23); and low/absent in three MAs (22–24) [Jiang et al., 2019: in PFS findings only (22)]. A sensitivity analysis to detect the source of heterogeneity was conducted in five MAs. None of these studies used other methods such as meta-regression for this purpose. Generally, heterogeneity did not have an excessive influence on the stability of the final pooled HRs (13, 14, 23). However, when excluding the study of Li et al. (33) from the MA of Jiang et al. (22), heterogeneity was dropped significantly for OS. PFS was not considerably affected. The publication bias was assessed based on Egger’s and Begg’s regression and rank correlation tests in two MAs (23, 24). Funnel plotting only was used in three MAs (13, 14, 27). One other MA assessed publication bias by Begg’s and Egger’s tests but included other cancer types (22). Overall, no publication bias was identified for both PFS and OS.

### Critical Appraisal of Included Systematic Reviews and Meta-Analyses

#### General Review

Of the 17 MAs evaluated (**Table 3**), only one has pre-registered its protocol on the PROSPERO database (30). Moreover, this MA does not have an updated record on this database despite its publication (PROSPERO accessed as of 01-07-2020). Reporting guidelines including Preferred Reporting Items for Systematic Reviews and Meta-Analyses (PRISMA) and Meta-analyses Of Observational Studies in Epidemiology (MOOSE) were used in only nine MAs. All included items have used the NOS for assessing the quality of non-randomized studies in MAs except for the one study by Ethier et al. (10). Twelve MAs stratified their findings using a subgroup analysis. Furthermore, two research teams used a subgroup analysis by one covariate only (14, 25), and two others did not provide it (20, 24). Funding was not acknowledged in three MAs (11, 24, 26). The gray literature was searched in 10 MAs but excluded earlier in the bibliographic research process, not stated in four MAs (11, 23, 26, 29) and not specified by two other authors (21, 24). In addition, the limitations of all included MAs were provided in the discussion of each publication.

**Table 3:** Qualitative assessment of appraised systematic reviews and meta-analyses.

Study	Registered on PROSPERO?	The reporting guideline used?^1^	NOS used?	Subgroup analysis provided?	Gray literature searched?	Funding acknowledged?	Meta-analysis limitations stated?
Cai et al., 2020 (19)	No	Yes (PRISMA)	Yes	Yes	Yes but excluded	Yes	Yes
Yin et al., 2019 (20)	No	No	Yes	No	Yes but excluded	No funding received	Yes
Gong et al., 2019 (8)	No	No	Yes	Yes	Yes but excluded	No funding received	Yes
Gao et al., 2019 (21)	No	Yes (PRISMA)	Yes	Yes	Not specified	Yes	Yes
Lu et al., 2019 (11)	No	No	Yes	Yes	Not stated	Not stated	Yes
Tian et al., 2018 (13)	No	No	Yes	Yes	Yes but excluded	No funding received	Yes
Xu et al., 2018 (23)	No	Yes (PRISMA)	Yes	Yes	Not stated	Yes	Yes
Zhao et al., 2018 (24)	No	Yes (PRISMA)	Yes	No	Not specified	Not stated	Yes
Chen et al., 2018 (25)	No	No	Yes	Yes (by one covariate only)	Yes but excluded	Yes	Yes
Zhu et al., 2018 (14)	No	Yes (PRISMA)	Yes	Yes (by one covariate only)	Yes but excluded	No funding received	Yes
Chen et al., 2017 (26)	No	No	Yes	Yes	Not stated	Not stated	Yes
Ma et al., 2017 (27)	No	No	Yes	Yes	Yes but excluded	Yes	Yes
Huang et al., 2017 (28)	No	Yes (MOOSE)	Yes	Yes	Yes but excluded	Yes	Yes
Yang et al., 2017 (29)	No	Yes (MOOSE)	Yes	Yes	Not stated	Yes	Yes
Ethier et al., 2017 (10)	No	Yes (PRISMA)	No	Yes	Yes but excluded	No funding received	Yes
Zhou et al., 2017 (30)	Yes (CRD42016052250)^#^	Yes (PRISMA)	Yes	Yes	Yes but excluded	Yes	Yes

NOS, Newcastle–Ottawa Scale; PRISMA, Preferred Reporting Items for Systematic Reviews and Meta-Analyses; MOOSE, Meta-analyses Of Observational Studies in Epidemiology.

^1^MOOSE or PRISMA.

^#^Not updated on PROSPERO database.

#### AMSTAR-2-Based Evaluation and Evidence Grading

The findings of the methodological assessment indicated that the quality of MAs was low in 11 of the 17 analyzed MAs (**Table 4**). Only six MAs were rated as of moderate quality. Nearly all included MAs did not register their review protocol on the PROSPERO database or other engines (Q2). Also, none of the MAs have explained the study designs for inclusion eligibility in their MAs (Q3). Likewise, the list of excluded studies with a justification of exclusion was not provided in all MAs (Q7). Furthermore, an explicit description of the included studies in each MA was observed in only five items (Q8). The risk of bias (RoB) assessment was absent in only one MA (Q9), and the source of funding in individual studies was not provided in all MAs (Q10). However, the potential impact of RoB on the pooled results in primary studies and their interpretation/discussion was not adequately evaluated (Q12 and Q13). Regarding evidence grading (**Table 5**), all MAs were downgraded to Class III or IV because of the retrospective design of included reports. Three items on LMR were graded as suggestive and one as of low evidence. Three items on NLR were graded as suggestive and two as weak evidence, and the remaining were not rated. Moreover, three items on PLR were graded as suggestive and one as weak evidence, and the remaining items were not rated.

**Table 4:** Critical appraisal of included meta-analyses based on AMSTAR-2 and evidence grading.

Study	Q1	Q2^‡^	Q3	Q4^‡^	Q5	Q6	Q7^‡^	Q8	Q9^‡^	Q10	Q11^‡^	Q12	Q13^‡^	Q14	Q15^‡^	Q16	AMSTAR-2 overall quality
Cai et al., 2020 (19)	Y	N	N	Y	Y	Y	N	PY	Y	N	Y	Y	N	Y	Y	Y	Low-quality review
Yin et al., 2019 (20)	Y	N	N	Y	Y	Y	N	PY	Y	N	Y	Y	N	Y	Y	Y	Low-quality review
Gong et al., 2019 (8)	Y	N	N	Y	N	Y	N	Y	Y	N	Y	Y	Y	N	N	Y	Moderate-quality review
Gao et al., 2019 (21)	Y	N	N	Y	Y	Y	N	Y	Y	N	Y	Y	Y	Y	Y	Y	Moderate-quality review
Lu et al., 2019 (11)	Y	N	N	Y	Y	Y	N	PY	Y	N	Y	Y	Y	Y	Y	N	Moderate-quality review
Tian et al., 2018 (13)	Y	N	N	Y	Y	Y	N	PY	Y	N	N	Y	Y	Y	Y	Y	Low-quality review
Xu et al., 2018 (23)	Y	N	N	PY	Y	N	N	N	Y	N	Y	N	N	Y	Y	Y	Low-quality review
Zhao et al., 2018 (24)	Y	N	N	Y	N	Y	N	N	Y	N	Y	Y	N	Y	Y	Y	Low-quality review
Chen et al., 2018 (25)	Y	N	N	Y	N	Y	N	PY	Y	N	Y	N	N	Y	Y	Y	Low-quality review
Zhu et al., 2018 (14)	Y	N	N	PY	Y	Y	N	PY	Y	N	Y	N	N	Y	Y	Y	Low-quality review
Chen et al., 2017 (26)	N	N	N	PY	N	Y	N	PY	Y	N	Y	N	N	N	Y	N	Low-quality review
Ma et al., 2017 (27)	Y	N	N	Y	Y	Y	N	Y	Y	N	Y	N	N	Y	Y	Y	Low-quality review
Huang et al., 2017 (28)	Y	N	N	PY	Y	Y	N	PY	Y	N	Y	N	N	Y	Y	Y	Low-quality review
Yang et al., 2017 (29)	Y	N	N	Y	Y	Y	N	PY	Y	N	Y	N	Y	Y	Y	Y	Moderate-quality review
Ethier et al., 2017 (10)	Y	N	N	Y	Y	Y	N	PY	N	N	Y	N	N	Y	Y	Y	Low-quality review
Zhou et al., 2017 (30)	Y	Y	N	Y	N	Y	N	Y	Y	N	Y	Y	Y	Y	Y	Y	Moderate-quality review

N, no; PY, partial yes; Y, yes.

AMSTAR-2 items: Q1: Did the research questions and inclusion criteria for the review include the components of PICO? Q2: Did the report of the review contain an explicit statement that the review methods were established prior to the conduct of the review, and did the report justify any significant deviations from the protocol? Q3: Did the review authors explain their selection of the study designs for inclusion in the review? Q4: Did the review authors use a comprehensive literature search strategy? Q5: Did the review authors perform study selection in duplicate? Q6: Did the review authors perform data extraction in duplicate? Q7: Did the review authors provide a list of excluded studies and justify the exclusions? Q8: Did the review authors describe the included studies in adequate detail? Q9: Did the review authors use a satisfactory technique for assessing the risk of bias (RoB) in individual studies that were included in the review? Q10: Did the review authors report on the sources of funding for the studies included in the review? Q11: If meta-analysis was performed, did the review authors use appropriate methods for statistical combination of results? Q12: If meta-analysis was performed, did the review authors assess the potential impact of RoB in individual studies on the results of the meta-analysis or other evidence synthesis? Q13: Did the review authors account for RoB in primary studies when interpreting/discussing the results of the review? Q14: Did the review authors provide a satisfactory explanation for, and discussion of, any heterogeneity observed in the results of the review? Q15: If they performed quantitative synthesis, did the review authors carry out an adequate investigation of publication bias (small study bias) and discuss its likely impact on the results of the review? Q16: Did the review authors report any potential sources of conflict of interest, including any funding they received for conducting the review?

^‡^Critical items in AMSTAR-2.

**Table 5:** Evidence grading of appraised meta-analyses.

Author/year	Endpoints	Number of cases >1,000	*p*-Value of overall effect^¥^	Heterogeneity (I^2^) < 50%	95% prediction interval excluding the null	No small-study effects^‡^	Design of included studies	Evidence grading
**Lymphocyte-to-monocyte ratio**								
Cai et al., 2020 (19)	OS	Yes	*p* < 0.001	No	Yes	Yes	Retrospective	III
	PFS	Yes	*p* < 0.001	Yes	Yes	Yes	Retrospective	III
Gong et al., 2019 (8)	OS	Yes	*p* < 0.001	No	Yes	Data not available	Retrospective	III
	PFS	Yes	*p* < 0.001	Yes	Yes	Data not available	Retrospective	III
Gao et al., 2019 (21)	OS	Yes	*p* < 0.001	No	Yes	Yes	Retrospective	III
	PFS	Yes	*p* < 0.001	Yes	Yes	Yes	Retrospective	III
Lu et al., 2019 (11)	OS	Yes	*p* < 0.01	No	Yes	Yes	Retrospective	IV
	PFS	Yes	*p* < 0.01	Yes	Yes	Conditions not met	Retrospective	IV
**Neutrophil-to-lymphocyte ratio**								
Yin et al., 2019 (20)	OS	Yes	*p* < 0.001	No	Yes	Data not available	Retrospective	III
	PFS	Yes	*p* < 0.001	Yes	Yes	Data not available	Retrospective	III
Zhao et al., 2018 (24)	OS	Yes	Data not available	No	Yes	Yes	Retrospective	NR
	PFS	Yes	Data not available	Yes	Yes	No	Retrospective	NR
Zhu et al., 2018 (14)	OS	Yes	Data not available	No	Yes	Data not available	Retrospective	NR
	PFS	Yes	Data not available	No	Yes	Data not available	Retrospective	NR
Chen et al., 2018 (25)	OS	Yes	*p* = 0.005	No	Yes	Yes	Retrospective	IV
	PFS	Yes	*p* = 0.001	No	Yes	Yes	Retrospective	IV
Chen et al., 2017 (26)	OS	Yes	*p* = 0.04	No	Yes	Yes	Retrospective	IV
	PFS	Yes	*p* = 0.004	No	Yes	Yes	Retrospective	IV
Huang et al., 2017 (28)	OS	Yes	Data not available	No	Yes	Yes	Retrospective	NR
	PFS	Yes	Data not available	No	Yes	Yes	Retrospective	NR
Yang et al., 2017 (29)	OS	Yes	Data not available	No	Yes	Yes	Retrospective	NR
	PFS	Yes	Data not available	No	Yes	Yes	Retrospective	NR
Ethier et al., 2017 (10)	OS	Yes	*p* < 0.001	No	Yes	Data not available	Retrospective	III
	PFS	Yes	*p* < 0.001	No	Yes	Data not available	Retrospective	III
Zhou et al., 2017 (30)	OS	Yes	*p* < 0.001	No	Yes	No	Retrospective	III
	PFS	Yes	*p* < 0.001	No	Yes	No	Retrospective	III
**Platelet-to-lymphocyte ratio**								
Jiang et al., 2019 (22)	OS	Yes	*p* = 0.000	No	Yes	Data not available	Retrospective	III
	PFS	Yes	*p* = 0.000	Yes	Yes	Data not available	Retrospective	III
Tian et al., 2018 (13)	OS	Yes	*p* < 0.001	No	Yes	Data not available	Retrospective	III
	PFS	Yes	*p* < 0.001	No	Yes	Data not available	Retrospective	III
Xu et al., 2018 (23)	OS	Yes	*p* = 0.000	Yes	Yes	Yes	Retrospective	III
	PFS	Yes	*p* < 0.001	Yes	Yes	Yes	Retrospective	III
Zhao et al., 2018 (24)	OS	Yes	Data not available	Yes	Yes	Yes	Retrospective	NR
	PFS	Yes	Data not available	Yes	Yes	Yes	Retrospective	NR
Zhu et al., 2018 (14)	OS	Yes	Data not available	No	Yes	Data not available	Retrospective	NR
	PFS	Yes	Data not available	No	Yes	Data not available	Retrospective	NR
Ma et al., 2017 (27)	OS	Yes	*p* < 0.01	No	Yes	Data not available	Retrospective	IV
	PFS	Yes	*p* < 0.01	No	Yes	Data not available	Retrospective	IV

NR, not rated; OS, overall survival; PFS, progression-free survival.

^¥^Copied as shown by the full-text articles.

^‡^Based on Egger’s test.

## Discussion

Briefly, according to our umbrella review, pretreatment high NLR and PLR, as well as low LMR, were all demonstrated to have an independent predictive value of poor survival outcomes in EOC in all reviewed MAs. Inflammation is well documented in cancer initiation and progression (5). The systemic inflammatory response based on single or multiple biomarkers was widely investigated in a remarkable number of cancers (35). This showed actionable findings as easy-to-use and less-expensive predictive and prognostic biomarkers for cancer survival and therapy response. However, the high heterogeneity in the included studies and their poor study designs limit the extrapolation of their conclusions in clinical practice.

Systematic reviews with/or without MAs are supposed to provide improved evidence on emerging topics to support the Grading of Recommendations Assessment, Development and Evaluation (GRADE) approach for a better implementation of international guidelines. However, with the rising race of the “publish or perish” era, many of the abovementioned articles are conducted without rigorous respect to methods of conducting this type of research. The majority of our examined MAs were published by Chinese researchers. The low quality of Chinese MAs was recently exposed. Indeed, a recent qualitative appraisal of more than 560 MAs from Chinese researchers found several concerns that negatively impact the value of these papers including the RoB, imprecision, publication bias, and inconsistency (36). In addition, pressure on young Chinese researchers to publish in internationally indexed journals is also an issue affecting the quality of the published MAs (37). Remarkably, a number of our included MAs that pooled outcomes on the same topic from the same study period were published in the same year. This massive production is unnecessary and may mislead and harm the prestige of such articles in the evidence-based medicine era (38).

Our umbrella review also found that prior protocol registration on the PROSPERO database was performed in only one item, and this is an important drawback of the included MAs. Without a doubt, protocol registration is associated with an increased quality of systematic reviews of interventions (39). Moreover, this will promote transparency, reduce the potential RoB, and, importantly, avoid redundant duplications (40). The status of the only registered MA in our umbrella review was not updated. This is also a recent tendency of MAs publishing worldwide. A recent analysis of this trend showed that only a few records’ statuses were up to date (41). Therefore, more serious follow-up and evaluations by journal editors and peer reviewers are awaited.

Regarding NOS, all items assessed the quality of non-randomized studies in MAs except for one study by Ethier et al. (10). Therefore, this is a good point for the MAs on the investigated topic. The NOS requires less tailoring and skills, and it is an easy-to-use tool (42). Thus, it should be employed in all MAs of observational studies. The gray literature in the MAs was excluded earlier in the literature search or not explored at all in other cases. This source of data is of high importance to find unpublished findings in peer-reviewed journals, particularly negative studies. In fact, excluding the gray literature may lead to publication biases (43). PRISMA- and MOOSE-based reporting guidelines were used in nine out of the 17 MAs. This suboptimal adherence is widely investigated in healthcare literature (44–47). Again, the application of these recommendations should be enhanced, and action is needed by medical journals throughout more appropriate editorial policies. Notably, a subgroup analysis was undertaken in a good number of the items, which have also transparently provided funding acknowledgment. Funding sources and conflicts of interest may affect and compromise the conclusions of the MAs and their quality. Financial and non-financial reporting of conflicts of interest in MAs is still suboptimal (48–50). However, given the observational nature of the included non-industry sponsored studies in the MAs of our umbrella review, the risk of this concern is limited.

Evidence hierarchy and synthesis in clinical sciences require a critical and qualitative review of the available evidence. For appraising MAs of randomized or non-randomized studies of healthcare interventions (or both), the AMSTAR-2 tool was developed for this purpose and is extensively used (15). The findings of our assessment showed a low quality in 11 out of the 17 reviewed MAs. This negative rating is largely due to the absence of critical domains in AMSTAR-2-related results including Q2, Q4, Q7, Q9, Q11, Q13, and Q15. In fact, a clear report of the prior development the literature review methodology was insufficient. On the other hand, a limited comprehensive foundation of the literature search strategy was also noticed. In addition, excluded studies from the systematic reviews and the reasons for exclusion were also not provided. Moreover, the RoB assessment in individual included studies and the discussion of the related results were lacking in some appraised systematic reviews. The remaining items were of moderate quality and had also various key critical flaws. In addition, the retrospective design of the meta-analyzed studies is a central weakness of these MAs. The evidence grading of the MAs reviewed was suggestive or weak for all eligible Mas, which is in line with the low quality demonstrated using AMSTAR-2. Of note, we found it difficult to grade the evidence because the *p*-values of overall effects and their exact values were not provided in some MAs.

Tumor-promoting inflammation is an enabling characteristic that fosters other signaling hallmarks of cancer cells (1). This inflammatory pathway can contribute to cancer capabilities by providing growth factors and cytokines to the tumor stroma to sustain proliferation, angiogenesis, activation of epithelial-to-mesenchymal transition, invasion, and metastasis, as well as inhibition of cell death programs (1). In EOC, inflammation is considered as an important factor that impacts the tumorigenesis of the fallopian tubes, which were recently suggested as the principal origins of ovarian carcinogenesis (51, 52). In addition to its involvement in the early steps of EOC, inflammation has a notable role in the late process of ovarian tumorigenesis. Moreover, inflammation and its mediators in EOC participate actively in the innate and adaptive immune response to eliminate cancer cells [nicely reviewed by Savant et al. (53)]. However, escape from this ability by malignant cells during chronic inflammation promoted by other cancer hallmarks enables apoptosis and immune surveillance evasion and therefore progression of EOC. Remarkably, it was recently demonstrated that inflammation is a key contributor of ovarian cancer cell seeding (54), thus making this hallmark a hot target for further research to improve outcomes of this women’s cancer. Either primary or metastatic, the tumor microenvironment (TME) of EOC hosts an important number of immune cell types (55). These cells, mainly dendritic cells, regulatory T cells, myeloid-derived suppressor cells, and M2 macrophages, are well known to have an immunosuppressive phenotype on effective antitumor immune cells such as natural killers (NKs) and CD4 and CD8 lymphocytes (55). This immune contexture of EOC TME, particularly high-grade serous cancers, is characterized by a different enrichment of tumor-infiltrating lymphocytes (TILs) according to the density of the tumor inflammation status. The density of TILs according to this concept can divide EOC into “hot” or non-inflamed “cold” tumors (56). Contrary to cold EOC, TME in hot EOC is illustrated by high density of TILs, principally CD8 T lymphocytes, mutated breast cancer gene (*BRCA*) cancer cells, and also an enriched signaling of immune suppression such as programmed death-ligand 1 (PD-L1), programmed cell death 1 (PD-1), and lymphocyte activation gene 3 (LAG-3) (56). The absence of these features was suggested to be associated with platinum-resistant disease, which is an aggressive subtype of EOC and also low response to immune-checkpoint blockade (57). The impact of TILs in patients with EOC has previously been widely investigated (58). A recent MA of 19 eligible studies with more than 6,000 high-grade serous EOC patients demonstrated that TILs are potential prognostic biomarkers for PFS and OS in this setting.

Turning this hallmark into biomarkers for predicting prognosis and therapy response for better patients’ stratification seems to be promising. The exploration of peripheral immune response in predicting survival of cancer patients has been extensively studied in EOC as discussed in this umbrella systematic review. Moreover, these immune biomarkers also emerged as potential predictors of outcomes in other solid cancers beyond EOC as suggested by multiple recent systematic reviews and MAs (59–62). However, whether these inexpensive circulating biomarkers based on peripheral blood correlate well with TILs or not is an area that has provided a rich literature and rationale for future well-conducted prospective studies. The predictive value of these inflammatory biomarkers based on complete blood cell count for therapy selection at diagnosis of EOC has also been evaluated. A previous retrospective cohort by Miao et al. showed that NLR and PLR predicted platinum resistance in EOC (63). Thus, confirming the earlier finding of other reports suggests their potential in predicting worse response to first-line platinum-based chemotherapy (34, 64). Recently, two biomarker analyses of the Italian MITO 24 group confirmed these findings and found that NLR at baseline correlates with sensitivity to platinum compounds and also to the antiangiogenic drug bevacizumab (65, 66). Methodologically, the assessment of these pretreatment systemic host responses at baseline can easily be performed for patients’ selection according to the results of these reports. Again, the retrospective nature of these studies cannot allow any recommendation for their use in clinical practice. In addition, another concern is the non-linearity of NLR observed in some cancer studies; therefore, the use of this variable as continuous or dichotomous during statistical analysis is questionable (67).

Promisingly, the combination of these cellular biomarkers with inflammatory mediators such as interleukin 6 (IL-6) and tumor necrosis factor alpha (TNF-α) as well other cytokines and proteomic biomarkers may increase the accuracy for predicting outcomes in EOC. IL-6 has been shown to stimulate pro-metastatic phenotype and also resistance to chemotherapy through Janus kinase (JAK) and signal transducer and activator of transcription 3 (STAT3) pathway (68). Furthermore, IL-6 pathway was found to be associated with chemoresistance in ovarian tumors, and their therapeutic targeting seems to enhance re-sensitization (69, 70). IL-6 in the peritoneal fluids of patients with EOC was shown to have a prognostic value. In a recent report by Rodrigues et al., IL-6 showed an independent association with OS (71). When combined with TNF-α, IL-6 predicted worse survival outcomes, suggesting an interaction between these two cytokines in driving progression and resistance to chemotherapy (72). Thus, the use of multimarker-based scores encompassing these inflammatory factors may enhance the power of previously established biomarkers such as CA-125 and human epididymis protein 4 (HE4) for EOC patients’ triage. It is hoped that future studies with prospective patients’ enrollment will include prognostic and predictive scores instead of single biomarkers to enhance the precision and improve accuracy.

Targeting these inflammatory pathways by anti-inflammatory drugs in treating EOC has been studied in several interventional clinical trials. The preclinical inhibition of cyclooxygenase 2 (COX-2), which is a key enzyme in the inflammatory cascade, yielded a promising reduction of the invasion capability of OC cells (73, 74). In a phase II trial, the use of oral celecoxib, a COX-2 inhibitor, in association with carboplatin showed a well-tolerated toxicity profile with a 28.9% overall response rate (75). When combined with metronomic cyclophosphamide, celecoxib did not improve survival outcomes in recurrent EOC (76). To date, this anti-inflammatory approach did not show clinical activity in EOC. Other ongoing clinical trials using combinatorial strategies associating celecoxib and aspirin with chemotherapy, immune-checkpoint inhibitors, and antiangiogenics may provide interesting results in the future (NCT02432378, NCT00538031, and NCT02659384). The use of inflammation as a druggable target and as a biomarker for outcomes in EOC will hold a promise if future studies focus more on providing strong study designs rather than reproduced the findings of the previous real-world cohorts.

To our best knowledge, this umbrella review is the first to systematically report, compile, and appraise the current clinical evidence on the prognostic value of inflammation-based biomarkers in EOC using AMSTAR-2. As for all research, our study has some limitations. First, the MAs reviewed have serious flaws in their design and therefore their findings. Moreover, despite no significant publication bias was detected in those MAs, the probability of intentional or unintentional exclusion of studies should be mentioned. Another important weakness of these MAs is their prolific character with few included recent studies. Most of them were redundant. In addition, we did not review the quality of the individual reports, because this should be the responsibility of the authors of these MAs. And finally, we did not use the Preferred Reporting Items for Overviews of Reviews (PRIOR) checklist for reporting the findings of our umbrella review because it is still under development (77).

## Conclusions and Recommendations for Future Authors

An important number of systematic reviews and MAs have been conducted to assess the prognostic value of immune-based biomarkers in peripheral blood of EOC patients. These inexpensive biomarkers are promising, but the available evidence is still of low quality. The RoB assessment using AMSTAR-2 was deficient in this appraised research. Most of the latest MAs were redundant and have several flaws in their methodology. Prospective studies are needed to increase the quality of the current evidence. Importantly, all future systematic reviews with MAs on this topic should 1) register their study protocol on PROSPERO (or other databases) before conducting this type of research; 2) include the findings of negative studies and also of non-English literature; 3) pre-plan subgroup analyses to provide stratified evidence on the studied research questions; 4) use the NOS (or other scores) to evaluate the quality of included non-randomized studies in their MAs and PRISMA (or MOOSE) to report their findings; 5) conduct a sensitivity analysis (and/or meta-regression) if heterogeneity was detected; 6) perform updated MAs only when there are sufficient new publications; and finally, 7) the journal editors should not accept redundant MAs with no new studies.

## Data Availability Statement

The original contributions presented in the study are included in the article/supplementary material. Further inquiries can be directed to the corresponding author.

## Author Contributions

KE developed the project idea. KE and OA contributed to writing and revising the proofs; they also provided reciprocal feedback and appraised the selected MAs. SA supervised the project. All authors contributed to the article and approved the submitted version.

## Author Disclaimer

The contents of this paper reflect the authors’ perspectives and not of their institutions of affiliation.

## Conflict of Interest

The authors declare that the research was conducted in the absence of any commercial or financial relationships that could be construed as a potential conflict of interest.

## Publisher’s Note

All claims expressed in this article are solely those of the authors and do not necessarily represent those of their affiliated organizations, or those of the publisher, the editors and the reviewers. Any product that may be evaluated in this article, or claim that may be made by its manufacturer, is not guaranteed or endorsed by the publisher.
